# Pairing Alpaca and Llama-Derived Single Domain Antibodies to Enhance Immunoassays for Ricin

**DOI:** 10.3390/antib6010003

**Published:** 2017-02-02

**Authors:** Kendrick B. Turner, Sabrina Hardy, Jinny L. Liu, Dan Zabetakis, P. Audrey Brozozog Lee, Ellen R. Goldman, George P. Anderson

**Affiliations:** 1 US Naval Research Laboratory, Center for Biomolecular Science and Engineering, 4555 Overlook Ave SW, Washington, DC 20375, USA; kendrick.turner@nrl.navy.mil (K.B.T.); jinny.liu@nrl.navy.mil (J.L.L.); daniel.zabetakis@nrl.navy.mil (D.Z.); 2 US Naval Research Laboratory, HBCU Internship Program, 4555 Overlook Ave SW, Washington, DC 20375, USA; brinahardy@yahoo.com; 3 Nova Research Inc., 1900 Elkin St., Suite 230, Alexandria, VA 22308, USA; plee142@su.edu

**Keywords:** camelid, llama, alpaca, single domain antibody, disulfide bond, thermal stability, ricin

## Abstract

Previously, our group isolated and evaluated anti-ricin single domain antibodies (sdAbs) derived from llamas, engineered them to further increase their thermal stability, and utilized them for the development of sensitive immunoassays. In work focused on the development of therapeutics, Vance et al. 2013 described anti-ricin sdAbs derived from alpacas. Herein, we evaluated the utility of selected alpaca-derived anti-ricin sdAbs for detection applications, and engineered an alpaca-derived sdAb to increase its melting temperature, providing a highly thermal stable reagent for use in ricin detection. Four of the alpaca-derived anti-ricin A-chain sdAbs were produced and characterized. All four bound to epitopes that overlapped with our previously described llama sdAbs. One alpaca sdAb, F6, was found to possess both a high melting temperature (73 °C) and to work optimally with a thermally stable llama anti-ricin sdAb in sandwich assays for ricin detection. We employed a combination of consensus sequence mutagenesis and the addition of a non-canonical disulfide bond to further enhance the thermal stability of F6 to 85 °C. It is advantageous to have a choice of recognition reagents when developing assays. This work resulted in defining an additional pair of highly thermal stable sdAbs for the sensitive detection of ricin.

## 1. Introduction

VHH, generally known as single domain antibodies (sdAbs), are variable domains from the heavy chain antibodies produced by members of the camelid family. That this family of animals possessed this unique class of immunoglobulins was first reported in the 1990s [1]. Since that time a growing number of sdAbs have been developed that recognize a wide variety of targets from small molecules to proteins, viruses, and bacteria [2,3,4]. There are a number of reasons that sdAbs are becoming increasingly utilized. For example, their small size permits them to be well expressed in *Escherichia coli (E.coli)*, alone or as fusion constructs with effector domains, i.e., alkaline phosphatase or rhizavidin, a biotin binding protein [5,6,7,8,9]. In addition, sdAbs are naturally more robust than conventional antibodies; most can be thermally or chemically denatured and can then refold to recover their binding activity [10,11]. It is their thermal stability that has been most attractive for their usage in analytic devices intended for operation in austere locations where the refrigeration required to properly store fragile immunoreagents is often lacking [12,13].

To enable biothreat detection devices that can function in austere environments, without undue logistical support, we and others have been developing sdAbs against a wide range of threat agents [2,14,15,16,17,18,19,20,21]. Ricin, a potential biothreat agent, is a 60-KDa protein toxin that consists of an A and B chain, both ~30 KDa. The ricin A chain has the ribosome inactivating activity, while the B chain is responsible for cell binding. Thus both halves are required for full toxicity. Ricin continues to be an agent of great concern due to its high toxicity, but even more so due to its ease of production from a readily available source, castor beans. Ricin is the unwanted byproduct of castor oil production, of which 600–800 million pounds are produced annually. While normally ricin’s toxicity is destroyed during the high heat of processing, a huge supply of raw material exists for the production of the toxin should someone so desire.

Ricin, a protein toxin, is typically detected via an immunoassay. Previously, we had developed sdAbs against ricin that recognized epitopes on the ricin A and B chains [22,23]. More recently, we developed variants of several of the ricin-binding sdAbs with improved thermal stabilities [24]. Although we were focused on developing reagents for detection, we showed that one family of sdAbs was able to neutralize ricin [22,25]. Members of a sequence family are highly homologous, having a nearly identical framework and complementarity determining regions (CDRs). Others, including Vance et al. have also developed sdAbs against ricin with the goal of achieving toxin neutralization [26,27]. The binders described by Vance et al. were isolated from immunized alpacas [26], while our binders were derived from immunized llamas. Perhaps more importantly, the alpacas and llamas were immunized with different immunogens, thus we speculated that the resulting sdAbs might recognize distinct epitopes. The goal of this work was to identify sdAbs derived from the alpaca that possessed both high affinity and thermal stability and functioned well with our current highly stable anti-ricin sdAbs, and then to stabilize the best candidate if necessary to provide an enhanced sandwich pair of binders that would enable operation in austere environments.

## 2. Results and Discussion

**Figure 1:**
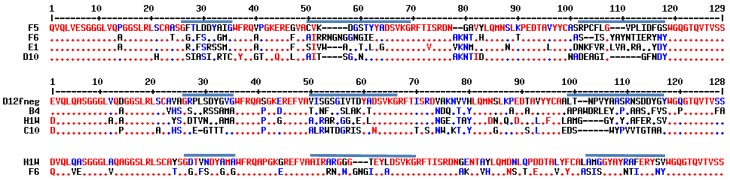
Protein sequences. Sequences of alpaca-derived single domain antibodies (sdAbs) shown on top [26], llama-derived sdAbs in the middle [23,24], and an alignment of H1W and F6 is shown on the bottom to allow comparison of their complementarity determining region (CDR) 3 sequences. CDRs 1, 2 and 3 are indicated by the solid line above the sequence; “.” denotes conserved sequence. Numbering shown is sequential from the N to C terminus.

To commence this work we chose four alpaca anti-ricin A-chain sdAbs (D10, E1, F5 and F6), which seemed likely to bind differing epitopes, based on the published data [26]. We had the genes for the four sdAbs synthesized commercially, and then we cloned them into the commercially available pET22b expression vector which includes a C-terminal 6×His tail. Cloning was confirmed by DNA sequencing and the sequence of these clones minus the amino acids due to the C-terminal restriction sites (AAALE) and the 6×His tail is shown in Figure 1. Previously, these sdAbs had been produced as recombinant thioredoxin fusion proteins containing an N-terminal 6×His tail and C-terminal E epitope tag (GAPVPYPDPLEPR) [26]. When we produced these proteins with only the 6×His tail, we found that three of the four produced very well. Yields of D10, E1, and F6 from two independent 500-mL shake flask production runs were 13.5 ± 1.5 mg/L, 12 ± 1.0 mg/L, and 17.5 ± 5.5 mg/L respectively. F5 was found consistently to be a poor producer, yielding only 0.9 ± 0.2 mg/L. This was likely due to the possible presence of a non-canonical second disulfide bond between CDR2 and CDR3; it is well known that presence of an additional non-canonical disulfide bond can result in decreased expression yields in *E. coli* [28,29,30].

We evaluated the sdAbs in terms of melting temperature by a fluorescence-based melting assay (dye melt) and circular dichroism (CD) methods and determined their refoldability by CD (Table 1). For comparison, we included two of our llama anti-ricin sdAbs (D12fneg and H1W) in these tests [24]. Three of four of the alpaca sdAbs displayed very good melting points as determined by CD, at or above 70 °C and refolded well (≥70%). Only E1 had a lower melting temperature, measured at 66 °C by CD.

We evaluated the affinity of the alpaca sdAbs against the ricin A chain (RTA) as well as their epitope specificity by surface plasmon resonance (SPR). These tests found that all four sdAbs had low-nM to sub-nM dissociation constants (K_D_s; Table 1 and Figure S1). The results for F5 and D10 were in good agreement with the K_D_s determined previously, being 0.28 nM and 0.11 nM, respectively [31]. The K_D_ determined for F6 binding RTA, on the other hand, was much higher than the 0.72-nM K_D_ determined previously for binding ricin [31]. However, this agrees with the observations reported by Vance et al that while the half maximal effective concentration to block activity (EC50) for D10 and F5 were unchanged for ricin and RTA, the EC50 value doubled for F6 [26], indicating F6 bound better to ricin than to RTA. Affinity constants for E1 were not previously reported.

We were interested in determining if the four alpaca-derived sdAbs bound independent or overlapping epitopes, and if they overlapped with any of the three epitopes on the ricin A chain we had identified for our llama-derived anti-ricin sdAbs. Utilizing SPR, we found that the alpaca sdAbs recognized two different epitopes and that these two epitopes overlapped the epitopes recognized by llama sdAbs (Figure S2). Alpaca sdAbs D10 and F5 overlapped each other and llama sdAb D12fneg. Of interest, D12fneg has been determined to inhibit ricin activity [25], just as both D10 and F5 were found to be protective.

In additional SPR competition studies it was observed that the alpaca-derived sdAbs E1 and F6 competed with each other as well as llama-derived sdAb H1W, suggesting that all three of these sdAbs bound to overlapping epitopes. In agreement with these results, there is significant sequence similarity in CDR3 between F6 and H1W sdAbs (Figure 1). In addition, F6 and H1W both show higher-affinity binding to ricin than to RTA (Figure S3.)

The llama sdAb family (D1\C10) that binds a third ricin A chain epitope, but does not bind to the immobilized ricin A chain [23,24] was not used in the SPR competition studies. However, it was included in a follow up enzyme-linked immunosorbent assay (ELISA) that confirmed the SPR results (Table S1). Here, C10 paired well with the other llama and alpaca sdAbs in a sandwich assays format, confirming that C10 recognizes a distinct epitope on ricin.

**Figure 2:**
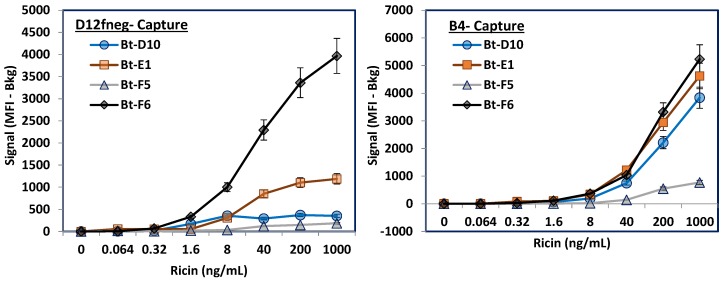
MagPlex evaluation of alpaca-derived anti-ricin sdAbs as tracers in sandwich assays in combination with two llama-derived anti-ricin sdAbs. The alpaca-derived sdAbs all recognize the ricin A chain. The capture D12fneg (**Left**) is specific for the ricin A chain, while the B4 capture (**Right**) binds to the ricin B chain. Only data from the relevant capture set is shown for each of the biotinylated (Bt) tracers; other capture sets present are not shown to facilitate visualization of the desired comparison. The signal shown is the median fluorescent intensity (MFI) obtained for that concentration of ricin minus the MFI in the absence of ricin. Error bars represent the standard error of the mean.

Next, we evaluated how the alpaca sdAbs paired with our llama sdAbs in sandwich immunoassays either using MagPlex assays or ELISA. For the MagPlex assays all the sdAbs were covalently attached to different sets of MagPlex microspheres as well as biotinylated (Bt). The sdAbs immobilized on the MagPlex microspheres serve to capture the ricin while the Bt-sdAbs are termed tracers and function as probes, or reporters, providing a route for signal generation through a dye-labeled streptavidin. Combinations of captures and tracers were evaluated for their ability to detect various concentrations of ricin. Figure 2 shows the results for Bt-alpaca sdAbs using either llama-derived anti-ricin sdAb D12fneg or B4 as the capture bead set. When D12fneg acted as the capture sdAb, Bt-F6 outperformed Bt-E1. As expected, Bt-D10 and Bt-F5 performed poorly as their binding had been shown to overlap with D12fneg. When utilizing the llama-derived ricin B-chain-binding sdAb B4 [23] as the capture, Bt-D10, Bt-E1, and Bt-F6 all performed similarly; only Bt-F5 did poorly. As F5’s epitope overlaps with D10 and D12fneg, the cause of this was not fully determined, but may relate to the fact it possesses a lysine residue in CDR2 that could lead to inactivation upon immobilization to the MagPlex microspheres or when biotinylated.

**Figure 3:**
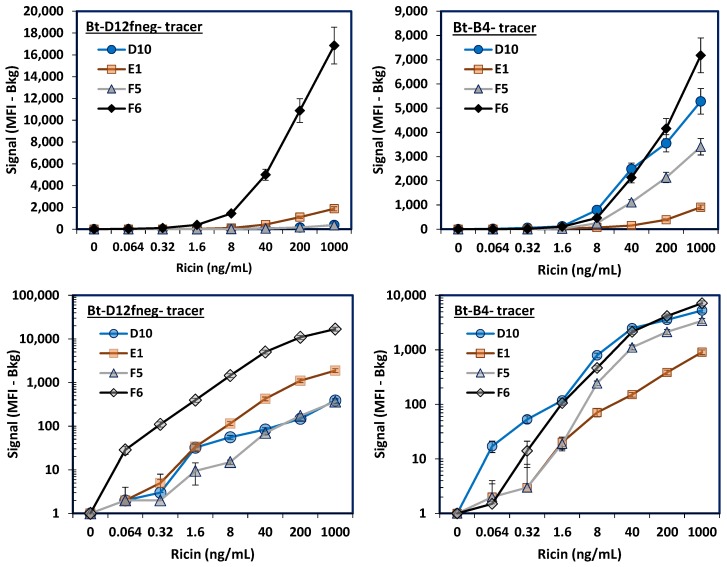
MagPlex evaluation of the alpaca-derived sdAbs as capture reagents combined with llama-derived anti-ricin sdAbs in sandwich assays for the detection of ricin. Top panels show signal on a linear scale, while the bottom two panels show a log scale. The D12fneg tracer (**Left Panels**) recognizes the ricin A chain, while the B4 tracer (**Right**) is specific for the ricin B chain. Error bars representing the standard error of the mean are shown for all the microsphere sets.

After testing the alpaca sdAbs as tracers, they were evaluated in MagPlex assays as the capture molecule. Figure 3 shows the results when either Bt-D12fneg or Bt-B4 were utilized as tracers; lower panels are log plots of the *y*-axis to better visualize results at the lower ricin concentrations. Alpaca-derived sdAb F6 paired exceptionally well with Bt-D12fneg, derived from llama, for the detection of ricin. This pair generated a robust signal, providing detection down to as low as 64 pg/mL; with a *p* = 0.00266, indicative of a highly significant difference. E1 did much more poorly and may have lost activity during the immobilization step, as it also performed poorly when Bt-B4 was used as a tracer. A lysine in E1’s CDR3 could contribute to its poor performance when immobilized on microspheres.

The ricin assay was also evaluated using Bt-D12fneg as the tracer and F6, D1, B4, and D12fneg as the capture molecules (Figure S4). Again, F6 outperformed the other capture molecules, although the B4 capture also performed well. Previously we had identified B4 as a component of a sensitive sandwich ELISA for ricin [23]. As expected, D12fneg did poorly when used as both capture and tracer in the same sandwich assay. Ricin is an A-B heterodimer without repeating epitopes, thus it is not expected that sdAbs will function well as both capture and tracer in the same assay.

**Figure 4:**
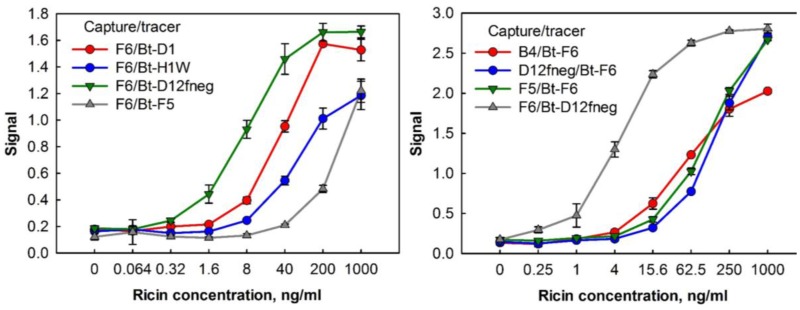
Sandwich enzyme-linked immunosorbent assays (ELISAs) using combinations of the alpaca- and llama-derived anti-ricin sdAbs. Left panel shows the alpaca-derived sdAb F6 paired with the alpaca-derived D1 and F5 reporters as well as the llama-derived H1W and D12fneg reporters. The right panel shows four different combinations of the anti-ricin sdAbs. Data points represent the average of triplicate measurements within one 96-well plate; error bars represent the standard deviation.

To examine how the F6 and Bt-D12fneg pair performed on a different assay platform, sandwich assays for ricin detection were performed by ELISA. Figure 4, compares the results of different sdAb capture and tracer pairs. The best detection was found with F6 as the capture molecule and Bt-D12fneg as the tracer. Thus, we demonstrated in a second assay format that by pairing one of the alpaca sdAbs with one of our llama sdAbs we were able to achieve a highly sensitive immunoassay for ricin.

While F6 displayed a good thermal stability, with a melting temperature of 73 °C, we subjected the sdAb to mutagenesis towards increasing its melting temperature. We had previously stabilized three of our llama-derived anti-ricin sdAbs through a combination of consensus sequence mutagenesis, the addition of negative charge, and by adding a non-canonical disulfide bond [24]. A similar approach, using these three components, was utilized with the alpaca-derived F6 sdAb. First, through comparison of the F6 sequence with the consensus sequence found in the majority of camelid VHH, we incorporated the change H83Y. Next we made the sdAb more negative by introducing the changes Q1E and G16E.

**Figure 5:**
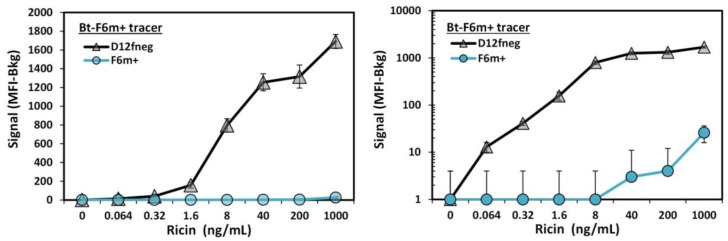
MagPlex evaluation of the thermal stabilized alpaca-derived F6m+ sdAb as the tracer reagent combined with llama-derived anti-ricin sdAb D12fneg in a sandwich assay for the detection of ricin. Left panels show signal on a linear scale, while the right panel shows a log scale. Error bars represent the standard error of the mean.

Finally we incorporated a second disulfide bond to the sdAb between framework regions by making the changes A49C and I73C, as first described by Hagihara et al. [32] and utilized by others [30,33,34]. The resultant clone, F6m+, possessed a melting temperature of 85 °C, an increase of 12 °C over the starting F6, and 83% refolding after heat denaturation as measured by CD. Addition of a non-canonical disulfide bond often leads to decreased expression yields in *E. coli* [28,29,30]; we did observe a decrease in yields from 17.5 ± 5.5 mg/L for F6 to 4.4 ± 2.1 mg/L for F6m+. Co-expression of chaperones may serve to increase the protein yields [35,36]. Some sdAbs show a decrease in affinity on the addition of a non-canonical disulfide bond [37]. This was the case for F6m+, where we measured the K_D_ to be 1.7 × 10^−9^ M for ricin binding which was approximately 5-fold higher than that measured for F6 (Figure S3). Yet when F6m+ was tested as a tracer in conjunction with the D12fneg capture in a MagPlex assay; it was still found to be highly effective (Figure 5). Thus, F6m+ represents a highly-stable ricin-binding sdAb that can be paired with our previously developed D12fneg clone for the sensitive detection of ricin.

In this work, a selection of anti-ricin sdAbs derived from an immunized alpaca were compared to sdAbs previously obtained from an immunized llama. We found the alpaca-derived sdAbs bound to the same or overlapping epitopes as anti-ricin sdAbs we had previously obtained. Having more clones to choose from, one can better assemble an immunoassay that provides the best combination of sensitivity and ruggedness of immunoreagents for applications in austere localities. By taking this approach, we successfully identified a pair of sdAbs against ricin, D12fneg and F6m+, which both yielded a highly sensitive assay (< 1 ng/mL of ricin) with reagents possessing remarkable thermal stability, at 79 and 85 °C, respectively. Future work will be to evaluate these identified reagents in assay formats being fielded in austere locations and determine shelf-life under harsh conditions.

## 3. Materials and Methods

### 3.1. Materials

The llama-derived anti-ricin sdAbs (B4, D12fneg, C10, and H1W) were previously described [22,23,24]. Ricin and ricin A chains (RTA) were purchased from Vector (Burlingame, CA, USA). Cloning enzymes were from New England Biolabs (Ipswich, MA, USA) and chemicals were from VWR (Radnor, PA, USA), or Sigma-Aldrich (St. Louis, MO, USA) unless otherwise indicated. Gene synthesis, and DNA sequencing were by Eurofins Genomics (Louisville, KY, USA). Sequence alignments were performed using MultAlin [38]. The full amino acid and DNA sequences for D12fneg and F6m+ are shown in Figure S5.

### 3.2. Protein Preparation

The alpaca anti-ricin sdAbs (D10, E1, F5 and F6) have been previously reported [26]. We had the genes for the four sdAbs synthesized with flanking NcoI and NotI sites for cloning into the pET22b(+) periplasmic expression vector. Similarly, the gene for the F6 variant, F6m+ was synthesized to include the changes Q1E, G16E, A49C, I73C, H83Y.

Each protein was prepared at least two times with cultures for each preparation started from independent single colonies. Replicate preparations were performed on different weeks and colonies were always started from fresh transformations.

Protein was produced essentially according to the protocol for periplasmic protein preparation described previously [23,28,39]. We transformed BL21(DE3) with pET22b-based expression plasmids, and grew colonies overnight at 37 °C on Luria broth (LB) agar plates with ampicillin (100 µg/mL). The next day, 50-mL overnight cultures were started from single colonies and grown at 25 °C in terrific broth (TB) with ampicillin (100 µg/mL). The overnight cultures were poured into 500 mL of TB with ampicillin and grown for a further 3 h at 25 °C. Expression was induced by addition of 0.5 mM isopropyl ß-d-1-thiogalactoside (IPTG), cultures were grown for an additional 2.5 h and the cells were pelleted. Cell pellets were first homogenized in 14 mL cold sucrose-tris (750 mM sucrose, 100 mM Tris pH 7.5), and then 28 mL of 1mM ethylenediaminetetraaceticacid (EDTA; pH 8) was added drop-wise to each sample. The cells were shaken for 15 min on ice before adding 1 mL of 500 mM MgCl_2_. Samples were then incubated on ice 10 min and the cells pelleted. Five mL of 10 × IMAC buffer (0.2 M Na_2_HPO_4_, 4 M NaCl, 0.2 M imidazole, pH 7.5) and 0.5 mL of Ni Separose (GE Healthcare, Pittsburgh, PA, USA) were added to the supernatant and the sample tumbled overnight at 4 °C on a rotisserie. The resin was washed twice in batch with 25 mL of 1 × IMAC buffer, poured into a small column, washed with ~10 mL 1 × IMAC buffer and finally eluted with 1 mL of 1 × IMAC buffer containing 250 mM imidazole. Protein was further purified into phosphate buffered saline (PBS) by size exclusion chromatography using a Superdex 75 10/300 GL column (GE Healthcare) and a Bio-Rad Duo-Flow System (Hercules, CA, USA). Yields of the sdAbs were determined by UV spectroscopy using a Nanodrop (Thermo Fisher, Waltham, MA, USA).

### 3.3. Circular Dichroism (CD)

As described previously, a Jasco J-815 CD spectrometer (Easton, MD, USA) was utilized to determine the melting temperature and refolding ability of the sdAbs [23,28,30]. Samples were diluted into deionized water to a final concentration of 40 µg/mL. As the temperature was increased from 25–90 °C at a rate of 2.5 °C/min, the differential absorbance of the sdAb sample was measured at 208 nm. The melting point correlated to the temperature at the inflection point between the folded and unfolded state. The error on the melting point determinations is within ±1 °C. For several of the constructs, replicate protein preparations were analyzed by CD and showed essentially the same melting and refolding behavior.

### 3.4. Fluorescence-Based Melting Assay

The melting temperature of each sdAb was measured by a fluorescent dye-based assay as outlined previously [24]. This technique relies on the fluorescence enhancement of Sypro Orange (Thermo Fisher), as it interacts with the hydrophobic amino acids on a protein, which become accessible upon thermal unfolding. Each sdAb was diluted to a concentration of 500 µg/mL in a final volume of 20 µL PBS. Then Sypro Orange dye was added to each sample at a dilution of 1:1000. Finally, samples were measured in triplicate using a StepOne Real-Time PCR machine (Applied Biosystems, Foster City, CA, USA). The heating program was run in continuous mode from 25–99 °C at a heating rate of 1% (~2 °C per minute), and data was recorded using the ROX filter. The melting point was determined to be the peak of the first derivative of the fluorescence intensity. All three replicates gave essentially identical values for the melting temperature.

### 3.5. Surface Plasmon Resonance (SPR)

Surface plasmon resonance (SPR) affinity and kinetics measurements were performed using the ProteOn XPR36 (Bio-Rad). Lanes of a general layer compact (GLC) chip were individually coated with ricin or ricin A chain. Immobilization of the proteins was performed using dilution to 20 µg/mL in 10 mM acetate buffer pH 5.0 and attached to the chip following the standard 1-ethyl-3-(3-dimethylaminopropyl)carbodiimide hydrochloride (EDC)/N-hydroxysulfosuccinimide (sulfo-NHS) coupling chemistry available from the manufacturer. Binding kinetics of each sdAb was tested at 25 °C by flowing six concentrations varying from 300 to 0 nM at 100 μL/min for 90 s over the antigen coated chip and then monitoring dissociation for 600 s. This generates binding data for each of the antigens immobilized on the chip. Following each run, the chip was regenerated by flowing 0.085% phosphoric acid (~pH 3.0) across the surface for 18 s. Data analysis was performed with ProteOn Manager 2.1 software, corrected by subtraction of the zero antibody concentration column as well as interspot correction. The standard error on the fits was less than 10%. Binding constants were determined using the Langmuir model built into the analysis software.

### 3.6. MagPlex Sandwich Immunoassays

MagPlex assays were performed essentially as described previously [23]. Briefly, MagPlex beads were coated with the desired sdAbs (D10, E1, F5, F6, B4 or D12fneg) using the recommended two step EDC/sulfo-NHS chemistry. The biotin-labeled sdAbs were prepared by using a 10-fold molar excess of NHS-LC-LC-biotin, after 30 min the excess biotin was removed using a Zeba spin 7 K desalting column (Thermo Fisher). The protein-coated MagPlex beads (~100/set) were mixed with various concentrations of ricin diluted into PBSTB (PBS + 0.05% Tween (PBST) and 1 mg/mL bovine serum albumin (BSA)) in the wells of a 96-well polystyrene round bottom microtiter plate. After 30 min the beads were washed by placing the plate on a 96f magnet (BioTek, Winooski, VT, USA) and washing three times with PBST. The beads were then incubated with the 1 µg/mL biotin labeled sdAb as indicated. After 30 min the beads were washed 3 times and then, to complete the fluorescent sandwich assay, the beads were incubated for 30 min with 2.5 µg/mL streptavidin conjugated phycoerythrin (SAPE, Columbia Biosciences, Frederick, MD, USA). After a final wash, the binding was measured on the MAGPIX instrument (Luminex Corp., Austin, TX, USA). The median fluorescent intensity (MFI) obtained by the evaluation of ≥50 microspheres for each set plotted, and error bars plotted as the standard error of the mean (SEM), which is typically less than ± 10% the mean, are plotted for all figures.

### 3.7. Enzyme-Linked Immunosorbent Assay

The sdAbs to be used for capture reagents were diluted to 1 ug/mL in PBS, and 100 µL added into wells of a 96-well plate (Nunc MaxiSorp, Thermo Fisher). Plates were securely covered with parafilm and incubated overnight at 4 °C. After washing the plate three times with PBST (PBS + 0.05% Tween 20) in the BioTek ELx50 washer, wells were blocked with PBSM (PBS + 4% powdered milk *w*/*v*), and incubated for one hour at room temperature. After washing the plate again three times, ricin diluted into 1% BSA in PBS (PBSB) was added to the sample wells and the plate incubated for one hour at room temperature. Wells were washed three times with PBST. Biotinylated sdAbs were diluted to a concentration of 1 µg/mL in PBSTB and added to a 100-µL/well. Plates were incubated a further hour at room temperature, and washed again. Streptavidin-conjugated horseradish peroxidase (diluted to 1 µg/mL in PBSTB) was added 100 µL/wells. Plates were incubated an hour at room temperature and washed with PBST. Signal was generated by adding 100 µL/well of Sure Blue TMB microwell Peroxidase substrate (KPL, Gaithersburg, MD, USA). After about five minutes, 100 µL/well of acid (2N HCl) was added to stop color development. Absorbance was read at 450 nm using the Tecan Infinite M1000 (Tecan, Research Triangle Park, NC, USA). Dose-response curves were collected using serial dilutions of ricin, starting at 1000 ng/mL. Experiments to measure the ability of the anti-ricin sdAbs to function as sandwich pairs utilized 250 ng/mL of ricin per well. In all cases, measurements were made in triplicate.

## Figures and Tables

**Table 1:** Affinity constants and melting temperatures.

SdAb	Melting Temperature		Affinity Constants to Ricin A Chain (RTA) by SPR		
	Dye Melt (°C)	CD (°C) (% Refold)	k_a_ (1/Ms)	k_d_ (1/s)	K_D_ (M)
D10 ^a^	66	70 (70%)	4.0 × 10^5^	5.7 × 10^−5^	1.4 × 10^−10^
E1 ^a^	65	66 (42%)	1.9 × 10^5^	2.4 × 10^−4^	1.3 × 10^−9^
F5 ^a^	70	71 (77%)	1.6 × 10^5^	2.8 × 10^−5^	1.7 × 10^−10^
F6 ^a^	74	73 (71%)	2.0 × 10^5^	1.2 × 10^−3^	6.0 × 10^−9^
D12fneg ^b^	77	79 (100%)	4.2 × 10^5^	9.4 × 10^−6^	2.3 × 10^−11^
H1W ^b^	70	71 (75%)	1.7 × 10^5^	3.4 × 10^−4^	2.0 × 10^−9^

^a^ Alpaca-derived sdAbs; ^b^ llama-derived sdAbs. CD: circular dichroism; k_a_: on rate; k_d:_ off rate; K_D_: dissociation constant; SPR: surface plasmon resonance

## Supplementary Materials

The following are available online at www.mdpi.com/2073-4468/6/1/3/s1, Figure S1: Determination of binding constants of various single domain antibodies (sdAbs) to the immobilized ricin A chain (RTA) by surface plasmon resonance (SPR) using a Bio-Rad ProteOn XPR36, Figure S2: Determination of epitope overlap by SPR competition binding to the ricin A chain, Figure S3: Binding affinities of F6, H1W and F6m+ to ricin, Table S1: Results of ELISA evaluation for pairing ability of anti-ricin sdAbs, Figure S4: MagPlex sandwich immunoassays with biotinylated (Bt)-D12fneg sdAb as the tracer, Figure S5: DNA and protein sequences for the final, optimized sdAbs, D12fneg and F6m+.

## References

[1] Hamers-Casterman C., Atarhouch T., Muyldermans S., Robinson G., Hamers C., Songa E.B., Bendahman N., Hamers R. (1993). Naturally-occurring antibodies devoid of light-chains. Nature.

[2] Eyer L.H.K. (2012). Single-domain antibody fragments derived from heavy-chain antibodies: A review. Vet. Med..

[3] Muyldermans S. (2013). Nanobodies: Natural single-domain antibodies. Annu. Rev. Biochem..

[4] De Marco A. (2011). Biotechnological applications of recombinant single-domain antibody fragments. Microb. Cell Fact..

[5] Swain M.D., Anderson G.P., Serrano-Gonzalez J., Liu J.L., Zabetakis D., Goldman E.R. (2011). Immunodiagnostic reagents using llama single domain antibody-alkaline phosphatase fusion proteins. Anal. Biochem..

[6] Walper S.A., Lee P.A.B., Goldman E.R., Anderson G.P. (2013). Comparison of single domain antibody immobilization strategies evaluated by surface plasmon resonance. J. Immunol. Methods.

[7] Liu J.L., Zabetakis D., Walper S.A., Goldman E.R., Anderson G.P. (2014). Bioconjugates of rhizavidin with single domain antibodies as bifunctional immunoreagents. J. Immunol. Methods.

[8] Sherwood L.J., Osborn L.E., Carrion R., Patterson J.L., Hayhurst A. (2007). Rapid assembly of sensitive antigen-capture assays for marburg virus, using in vitro selection of llama single-domain antibodies, at biosafety level 4. J. Infect. Dis..

[9] Liu X., Xu Y., Wan D.-B., Xiong Y.-H., He Z.-Y., Wang X.-X., Gee S.J., Ryu D., Hammock B.D. (2015). Development of a nanobody–alkaline phosphatase fusion protein and its application in a highly sensitive direct competitive fluorescence enzyme immunoassay for detection of ochratoxin a in cereal. Anal. Chem..

[10] Dumoulin M., Conrath K., van Meirhaeghe A., Meersman F., Heremans K., Frenken L.G.J., Muyldermans S., Wyns L., Matagne A. (2002). Single-domain antibody fragments with high conformational stability. Protein Sci..

[11] Van der Linden R.H.J., Frenken L.G.J., de Geus B., Harmsen M.M., Ruuls R.C., Stok W., de Ron L., Wilson S., Davis P., Verrips C.T. (1999). Comparison of physical chemical properties of llama V-HH antibody fragments and mouse monoclonal antibodies. Biochim. Biophys. Acta Protein Struct. Mol. Enzymol..

[12] Leski T.A., Ansumana R., Taitt C.R., Lamin J.M., Bangura U., Lahai J., Mbayo G., Kanneh M.B., Bawo B., Bockarie A.S. (2015). Use of filmarray™ system for detection of *Zaire ebolavirus* in a small hospital, Bo, Sierra Leone. J. Clin. Microbiol..

[13] Leski T.A., Ansumana R., Malanoski A.P., Jimmy D.H., Bangura U., Barrows B.R., Alpha M., Koroma B.M., Long N.C., Sundufu A.J. (2012). Leapfrog diagnostics: Demonstration of a broad spectrum pathogen identification platform in a resource-limited setting. Health Res. Policy Syst..

[14] Conway J.O., Sherwood L.J., Collazo M.T., Garza J.A., Hayhurst A. (2010). Llama single domain antibodies specific for the 7 botulinum neurotoxin serotypes as heptaplex immunoreagents. PLoS ONE.

[15] Sherwood L.J., Hayhurst A. (2013). Ebolavirus nucleoprotein C-termini potently attract single domain antibodies enabling monoclonal affinity reagent sandwich assay (MARSA) formulation. PLoS ONE.

[16] Walper S.A., Anderson G.P., Lee P.A.B., Glaven R.H., Liu J.L., Bernstein R.D., Zabetakis D., Johnson L., Czarnecki J.M., Goldman E.R. (2012). Rugged single domain antibody detection elements for bacillus anthracis spores and vegetative cells. PLoS ONE.

[17] Walper S.A., Lee P.A.B., Anderson G.P., Goldman E.R. (2013). Selection and characterization of single domain antibodies specific for bacillus anthracis spore proteins. Antibodies.

[18] Alzogaray V., Danquah W., Aguirre A., Urrutia M., Berguer P., Vescovi E.G., Haag F., Koch-Nolte F., Goldbaum F.A. (2011). Single-domain llama antibodies as specific intracellular inhibitors of SpvB, the actin ADP-ribosylating toxin of salmonella typhimurium. FASEB J..

[19] Stone E., Hirama T., Chen W.X., Soltyk A.L., Brunton J., MacKenzie C.R., Zhang J.B. (2007). A novel pentamer versus pentarner approach to generating neutralizers of verotoxin 1. Mol. Immunol..

[20] Dong J., Thompson A.A., Fan Y., Lou J., Conrad F., Ho M., Pires-Alves M., Wilson B.A., Stevens R.C., Marks J.D. (2010). A single-domain llama antibody potently inhibits the enzymatic activity of botulinum neurotoxin by binding to the non-catalytic alpha-exosite binding region. J. Mol. Biol..

[21] Wesolowski J., Alzogaray V., Reyelt J., Unger M., Juarez K., Urrutia M., Cauerhff A., Danquah W., Rissiek B., Scheuplein F. (2009). Single domain antibodies: Promising experimental and therapeutic tools in infection and immunity. Med. Microbiol. Immunol..

[22] Anderson G.P., Liu J.L., Hale M.L., Bernstein R.D., Moore M., Swain M.D., Goldman E.R. (2008). Development of antiricin single domain antibodies toward detection and therapeutic reagents. Anal. Chem..

[23] Anderson G.P., Bernstein R.D., Swain M.D., Zabetakis D., Goldman E.R. (2010). Binding kinetics of antiricin single domain antibodies and improved detection using a B chain specific binder. Anal. Chem..

[24] Turner K.B., Liu J.L., Zabetakis D., Lee A.B., Anderson G.P., Goldman E.R. (2015). Improving the biophysical properties of anti-ricin single-domain antibodies. Biotechnol. Rep..

[25] Legler P.M., Compton J.R., Hale M.L., Anderson G.P., Olson M.A., Millard C.B., Goldman E.R. (2017). Stability of isolated antibody-antigen complexes as a predictive tool for selecting toxin neutralizing antibodies. mAbs.

[26] Vance D.J., Tremblay J.M., Mantis N.J., Shoemaker C.B. (2013). Stepwise engineering of heterodimeric single domain camelid vhh antibodies that passively protect mice from ricin toxin. J. Biol. Chem..

[27] Shuntao W., Jiannan F., Jianwei G., Leiming G., Yan L., Yingxun S., Weisong Q., Meiru H., Gencheng H., Beifen S. (2006). A novel designed single domain antibody on 3-D structure of ricin a chain remarkably blocked ricin-induced cytotoxicity. Mol. Immunol..

[28] Walper S.A., Liu J.L., Zabetakis D., Anderson G.P., Goldman E.R. (2014). Development and evaluation of single domain antibodies for vaccinia and the L1 antigen. PLoS ONE.

[29] Liu J., Goldman E., Zabetakis D., Walper S., Turner K., Shriver-Lake L., Anderson G. (2015). Enhanced production of a single domain antibody with an engineered stabilizing extra disulfide bond. Microb. Cell Fact..

[30] Zabetakis D., Olson M.A., Anderson G.P., Legler P.M., Goldman E.R. (2014). Evaluation of disulfide bond position to enhance the thermal stability of a highly stable single domain antibody. PLoS ONE.

[31] Herrera C., Tremblay J.M., Shoemaker C.B., Mantis N.J. (2015). Mechanisms of ricin toxin neutralization revealed through engineered homodimeric and heterodimeric camelid antibodies. J. Biol. Chem..

[32] Hagihara Y., Mine S., Uegaki K. (2007). Stabilization of an immunoglobulin fold domain by an engineered disulfide bond at the buried hydrophobic region. J. Biol. Chem..

[33] Hagihara Y., Saerens D. (2014). Engineering disulfide bonds within an antibody. Biochim. Biophys. Acta Proteins Proteom..

[34] Saerens D., Conrath K., Govaert J., Muyldermans S. (2008). Disulfide bond introduction for general stabilization of immunoglobulin heavy-chain variable domains. J. Mol. Biol..

[35] Schlapschy M., Grimm S., Skerra A. (2006). A system for concomitant overexpression of four periplasmic folding catalysts to improve secretory protein production in escherichia coli. Protein Eng. Des. Sel..

[36] Shriver-Lake L.C., Goldman E.R., Zabetakis D., Anderson G.P. (2017). Improved production of single domain antibodies with two disulfide bonds by co-expression of chaperone proteins in the *Escherichia coli* periplasm. J. Immunol. Methods.

[37] Hussack G., Hirama T., Ding W., MacKenzie R., Tanha J. (2011). Engineered single-domain antibodies with high protease resistance and thermal stability. PLoS ONE.

[38] Corpet F. (1988). Multiple sequence alignment with hierarchical-clustering. Nucleic Acids Res..

[39] Goldman E.R., Brozozog-Lee P.A., Zabetakis D., Turner K.B., Walper S.A., Liu J.L., Anderson G.P. (2014). Negative tail fusions can improve ruggedness of single domain antibodies. Protein Expr. Purif..

