# Supplementary material for: Pairing Alpaca and Llama-Derived Single Domain Antibodies to Enhance Immunoassays for Ricin
Source: Antibodies (Basel). 2017 Feb 2;6(1):3. doi: 10.3390/antib6010003 (PMC6698814; doi:10.3390/antib6010003)
Supplement: Supplementary file 1 [file antibodies-06-00003-s001.docx]

Supplementary Materials: Pairing Alpaca and Llama-Derived Single Domain Antibodies to Enhance Immunoassays for Ricin

Kendrick B. Turner, Sabrina Hardy, Jinny L. Liu, Dan Zabetakis, P. Audrey Brozozog Lee,
Ellen R. Goldman and George P. Anderson

**Figure S1.** Determination of binding constants of various single domain antibodies (sdAbs) to the immobilized ricin A chain (RTA) by surface plasmon resonance (SPR) using a Bio-Rad ProteOn XPR36.

**Figure S2.** Determination of epitope overlap by SPR competition binding to ricin A chain. Panels **A** through **D** show examples of the epitope determination. Panel **A** tests for competition between D12fneg and F6, F5, and E1; results indicates only F5 competes with D12f as the blue line is much lower than the green line. Panel **B** tests for competition between D12fneg and D10 as well as H1W with F6 and F5; results indicate D12f competes with D10 as the red line is much lower than the light blue line, while H1W competes with F6 (blue line lower than green line) but not F5. Panel **C** tests for the competition between D10 and E1, F5, and F6; results indicate that D10 competes with F5 (blue line lower than green line) but not E1 or F6. Panel **D** tests for the competition between F6 and E1, F5, and H1W; results indicate the F6 competes with E1 (red line lower than light blue) and H1W (magenta line lower than orange line).

**Figure S3.** Binding affinities of H1W, F6, and F6m+ to ricin. Both H1W and F6 bind with higher affinity to ricin than to RTA (refer to Figure S1). The affinity of F6m+ is reduced by a factor of 5 versus F6.

**Table S1.** Results of ELISA evaluation for pairing ability of anti-ricin sdAb *.

| **Captures** | **Tracers** | | |
| --- | --- | --- | --- |
|  | **C10** | **D12fneg** | **H1W** |
| D10 | ++ | - | + |
| E1 | ++ | ++ | - |
| F5 | ++ | - | ++ |
| F6 | ++ | ++ | - |
| B4 | ++ | ++ | + |
| C10 | - | ++ | ++ |
| D12fneg | ++ | - | ++ |
| H1W | ++ | - | - |

***** The signal generated by the same sdAbs when tested as both capture and tracer was considered to be background. All the other values were divided by this background value. Values less than 1.2 were labeled (-), values ≥ 1.2 but < 2 were labeled (+), and values ≥ 2 were labeled (++).

**Figure S4.** MagPlex sandwich immunoassays with biotinylated (Bt)-D12fneg sdAbs as the tracer. Error bars representing the standard error of the mean are shown for each microsphere set.


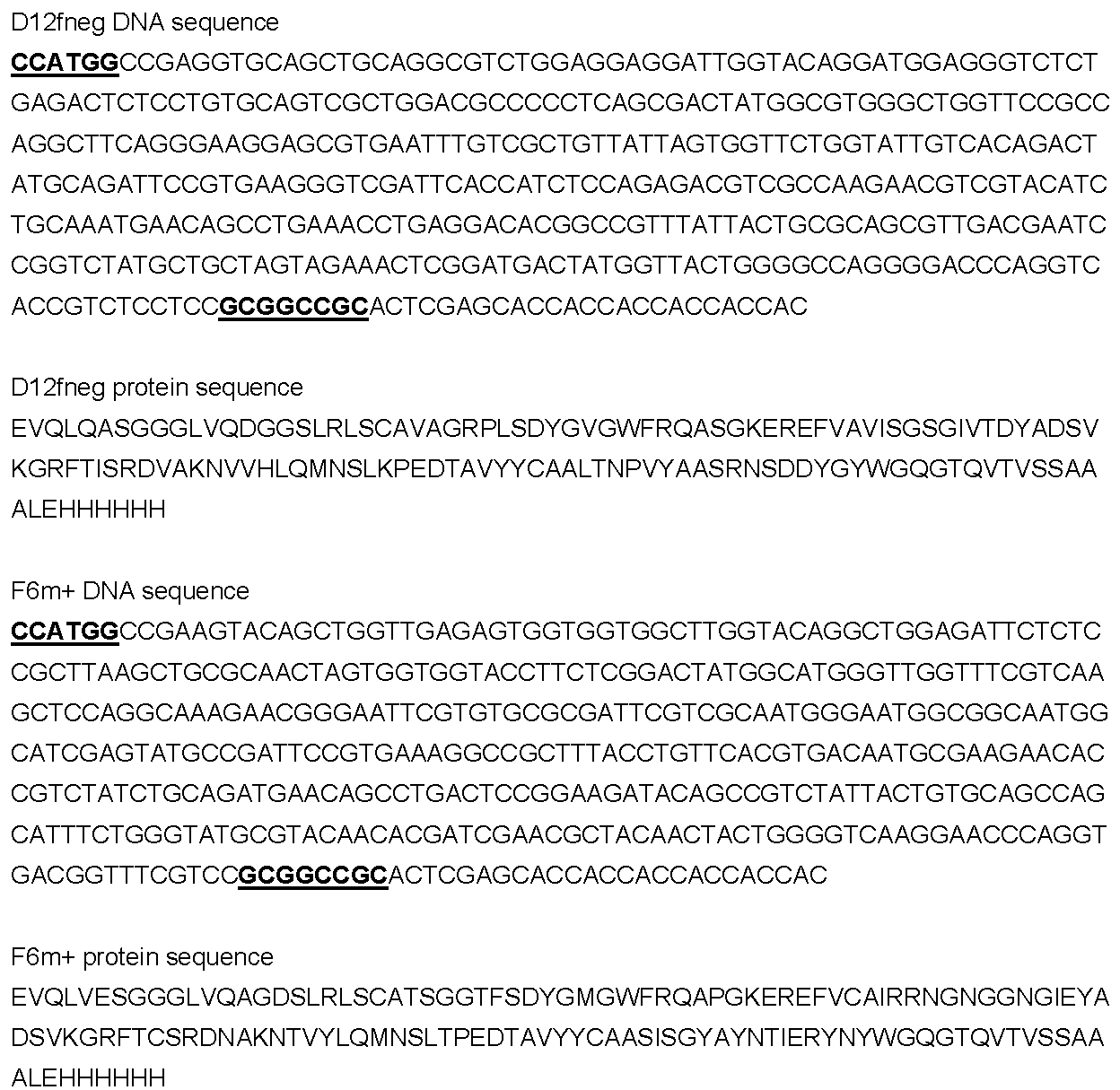


**Figure S5.** DNA and protein sequences for the final, optimized sdAbs, D12fneg and F6m+. The sdAbs are cloned into the commercial expression vector pet22b+ between the NcoI and NotI sites (sites underlined and bold).
